# A preoperative radiogenomic model based on quantitative heterogeneity for predicting outcomes in triple-negative breast cancer patients who underwent neoadjuvant chemotherapy

**DOI:** 10.1186/s40644-024-00746-z

**Published:** 2024-07-30

**Authors:** Jiayin Zhou, Yansong Bai, Ying Zhang, Zezhou Wang, Shiyun Sun, Luyi Lin, Yajia Gu, Chao You

**Affiliations:** 1 Department of Radiology, Fudan University Shanghai Cancer Center, No. 270 Dong’an Road, Shanghai, 200032 China; 2 Department of Oncology, Shanghai Medical College, Fudan University, Shanghai, 200032 China; 3 Institute of Science and Technology for Brain-Inspired Intelligence, Fudan University, Shanghai, 200000 China; 4 Department of Breast Surgery, Fudan University Shanghai Cancer Center, Shanghai, 200032 China; 5 Department of Cancer Prevention, Fudan University Shanghai Cancer Center, Shanghai, 200032 China; 6 Shanghai Municipal Hospital Oncological Specialist Alliance, Shanghai, 200000 China

**Keywords:** Breast cancer, Radiogenomics, Spatial heterogeneity, Pathological complete response, Prognosis

## Abstract

**Background:**

Triple-negative breast cancer (TNBC) is highly heterogeneous, resulting in different responses to neoadjuvant chemotherapy (NAC) and prognoses among patients. This study sought to characterize the heterogeneity of TNBC on MRI and develop a radiogenomic model for predicting both pathological complete response (pCR) and prognosis.

**Materials and methods:**

In this retrospective study, TNBC patients who underwent neoadjuvant chemotherapy at Fudan University Shanghai Cancer Center were enrolled as the radiomic development cohort (*n* = 315); among these patients, those whose genetic data were available were enrolled as the radiogenomic development cohort (*n* = 98). The study population of the two cohorts was randomly divided into a training set and a validation set at a ratio of 7:3. The external validation cohort (*n* = 77) included patients from the DUKE and I-SPY 1 databases. Spatial heterogeneity was characterized using features from the intratumoral subregions and peritumoral region. Hemodynamic heterogeneity was characterized by kinetic features from the tumor body. Three radiomics models were developed by logistic regression after selecting features. Model 1 included subregional and peritumoral features, Model 2 included kinetic features, and Model 3 integrated the features of Model 1 and Model 2. Two fusion models were developed by further integrating pathological and genomic features (PRM: pathology-radiomics model; GPRM: genomics-pathology-radiomics model). Model performance was assessed with the AUC and decision curve analysis. Prognostic implications were assessed with Kaplan‒Meier curves and multivariate Cox regression.

**Results:**

Among the radiomic models, the multiregional model representing multiscale heterogeneity (Model 3) exhibited better pCR prediction, with AUCs of 0.87, 0.79, and 0.78 in the training, internal validation, and external validation sets, respectively. The GPRM showed the best performance for predicting pCR in the training (AUC = 0.97, *P* = 0.015) and validation sets (AUC = 0.93, *P* = 0.019). Model 3, PRM and GPRM could stratify patients by disease-free survival, and a predicted nonpCR was associated with poor prognosis (*P* = 0.034, 0.001 and 0.019, respectively).

**Conclusion:**

Multiscale heterogeneity characterized by DCE-MRI could effectively predict the pCR and prognosis of TNBC patients. The radiogenomic model could serve as a valuable biomarker to improve the prediction performance.

**Supplementary Information:**

The online version contains supplementary material available at 10.1186/s40644-024-00746-z.

## Introduction

Triple-negative breast cancer (TNBC) has a poor prognosis, and effective therapeutic targets are lacking [1, 2]. Neoadjuvant chemotherapy (NAC) has been widely used as a first-line treatment for locally advanced TNBC, and pathological complete response (pCR) can be achieved in approximately one-third of patients [3–5]. A pCR after NAC is associated with improved disease-free and overall survival [6–10]. However, the long-term prognosis of some patients who have achieved pCR is still unsatisfactory [8–10]. To make appropriate treatment and surgical decisions, early and accurate prediction of both pCR and patient prognosis is of great clinical significance.

MRI does not have the harm of radiation, which could realize the dynamic monitoring during the tumor treatment process. Radiomics is a noninvasive technique that can reflect the overall characteristics of a tumor. In recent years, the emergence of new methods such as habitat imaging and peritumoral radiomics has demonstrated the potential of image-based characterization of tumor heterogeneity. Therefore, researchers are no longer limited to the analysis of the tumor body but rather to broadening the focus to the intratumor, peritumor and even entire background parenchyma of the breast. Recent studies on the tumor body have shown that radiomic features can quantify intratumoral spatial heterogeneity [11–16]. For the peritumoral regions, studies have shown that radiomic features can characterize the heterogeneity of the microenvironment around the tumor [17–22]. TNBC is a highly heterogeneous subtype, and simplifying the tumor into a single whole ignores spatial heterogeneity [23–25]. To comprehensively reflect the heterogeneity of the tumor and peritumoral parenchyma, we analyzed the radiomic features of the tumor body, subregions and peritumoral region.

Radiomics reflects the characteristics of tumors from a macroscopic perspective but may not be able to accurately reveal the biological nature of tumors. Genomic analysis requires acquiring a sample of tissue, which is invasive for the patient, but it reveals the heterogeneity of tumors more precisely at the molecular level. The integration of complementary data generated by radiomics and genomics may facilitate precision medicine and improve prognosis [26]. Radiogenomics uncovers the biological significance of radiomics by linking radiomics features to the genetic spectrum [26, 27]. A previous study focused on the relationship between MR image and the expression of breast cancer genes and revealed that MRI features were correlated with the expression of genes related to metastasis, drug resistance and prognosis [28]. A previous study by our team integrated MRI and genomic features and found that the radiogenomics model (AUC = 0.87; *P* = 0.04) demonstrated superiority in predicting pCR of TNBC compared to the radiomics model [29]. However, there are still few studies integrating radiomics and genomic features. We hope that on the basis of these previous studies, we can further develop a radiogenomic model based on multiregional radiomic features to improve the prediction performance.

Consequently, the purpose of this study was to characterize the multiscale heterogeneity of TNBC by multiregional MRI, develop a radiomic model for predicting pCR, and further integrate radiomic, clinicopathological and genomic features to develop a radiogenomic model to more effectively predict both pCR and prognosis.

## Materials and methods

### Patients

**Fig. 1:**
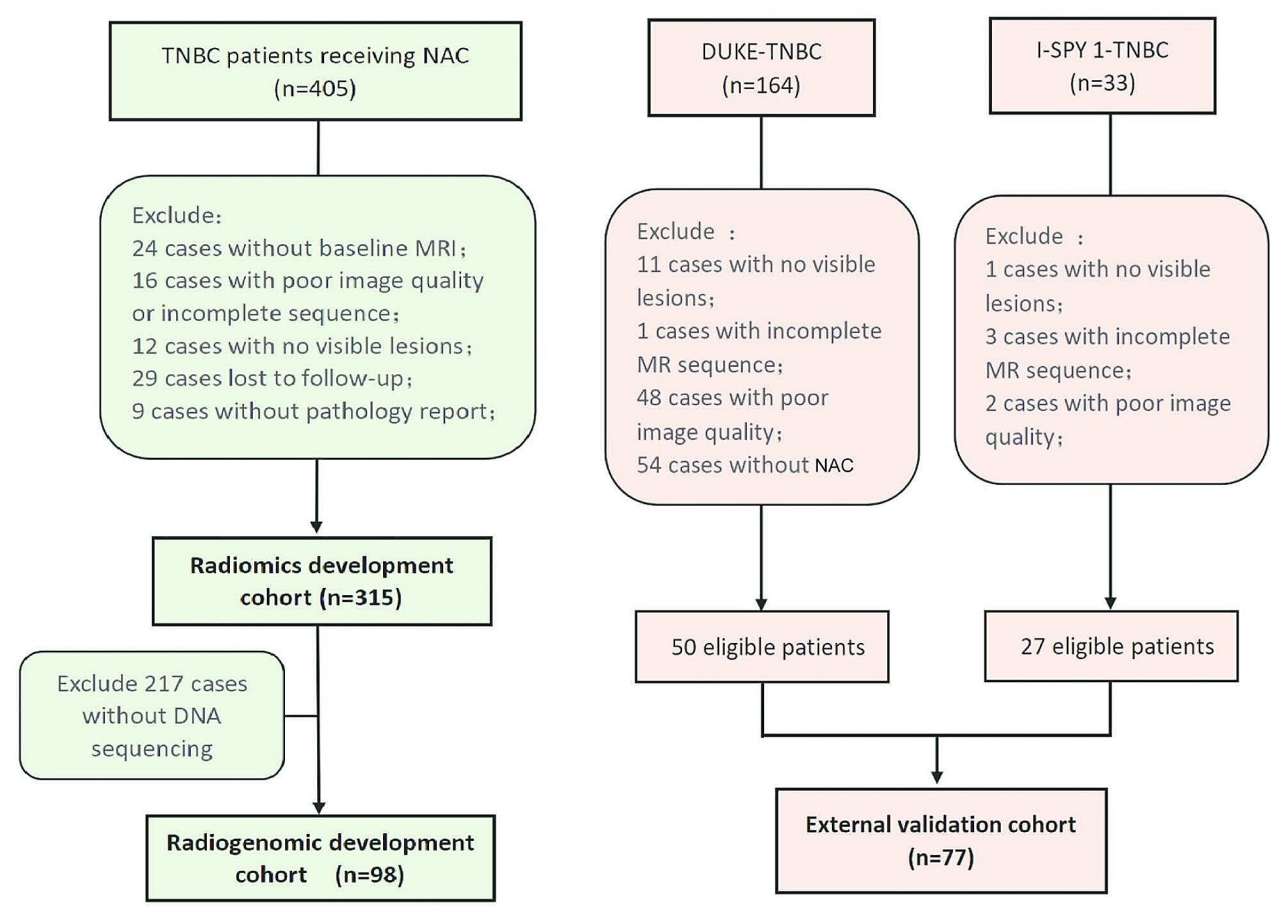
Flowchart of patient selection. TNBC patients receiving NAC = patients with triple-negative breast cancer receiving neoadjuvant therapy at our center; DUKE-TNBC = patients with triple-negative breast cancer from the DUKE dataset; I-SPY 1-TNBC = patients with triple-negative breast cancer from the I-SPY 1 dataset

The study was approved by the Ethics Committee of the Institutional Review Board (IRB) of our institution, and the requirement for patient informed consent was waived. In this retrospective study, female patients treated at Fudan University Shanghai Cancer Center from August 2011 to March 2022 were enrolled as the radiomics development cohort (*n* = 315). The inclusion criteria were as follows: (1) patients who were negative for the estrogen receptor (ER), progesterone receptor (PR) and human epidermal growth factor receptor 2 (HER2) according to a core-needle biopsy performed before treatment (the HER2 score 2 + obtained based on immunohistochemistry and gene amplification was confirmed with fluorescence in situ hybridization) and (2) patients who received NAC and eventually underwent surgery. The exclusion criteria were as follows: (1) patients lacking baseline dynamic contrast-enhanced (DCE) MR images; (2) patients with poor-quality or incomplete MR images; (3) patients with no visible lesions; (4) patients without final pathological results after treatment; and (5) patients lost to follow-up. Patients with available DNA sequencing data were selected to form the radiogenomic development cohort (*n* = 98). The study population was randomly divided into a training set and a validation set at a ratio of 7:3. In the radiomics development cohort, there were 223 patients in the training set and 92 patients in the validation set. In the radiogenomic development cohort, there were 69 patients in the training set and 29 patients in the validation set. Patients from the DUKE dataset and the I-SPY 1 dataset were used as the external validation cohort (*n* = 77) for the radiomic models. The detailed inclusion/exclusion criteria are shown in Additional file 1. The enrollment process is shown in Fig. 1.

### Study design

**Fig. 2:**
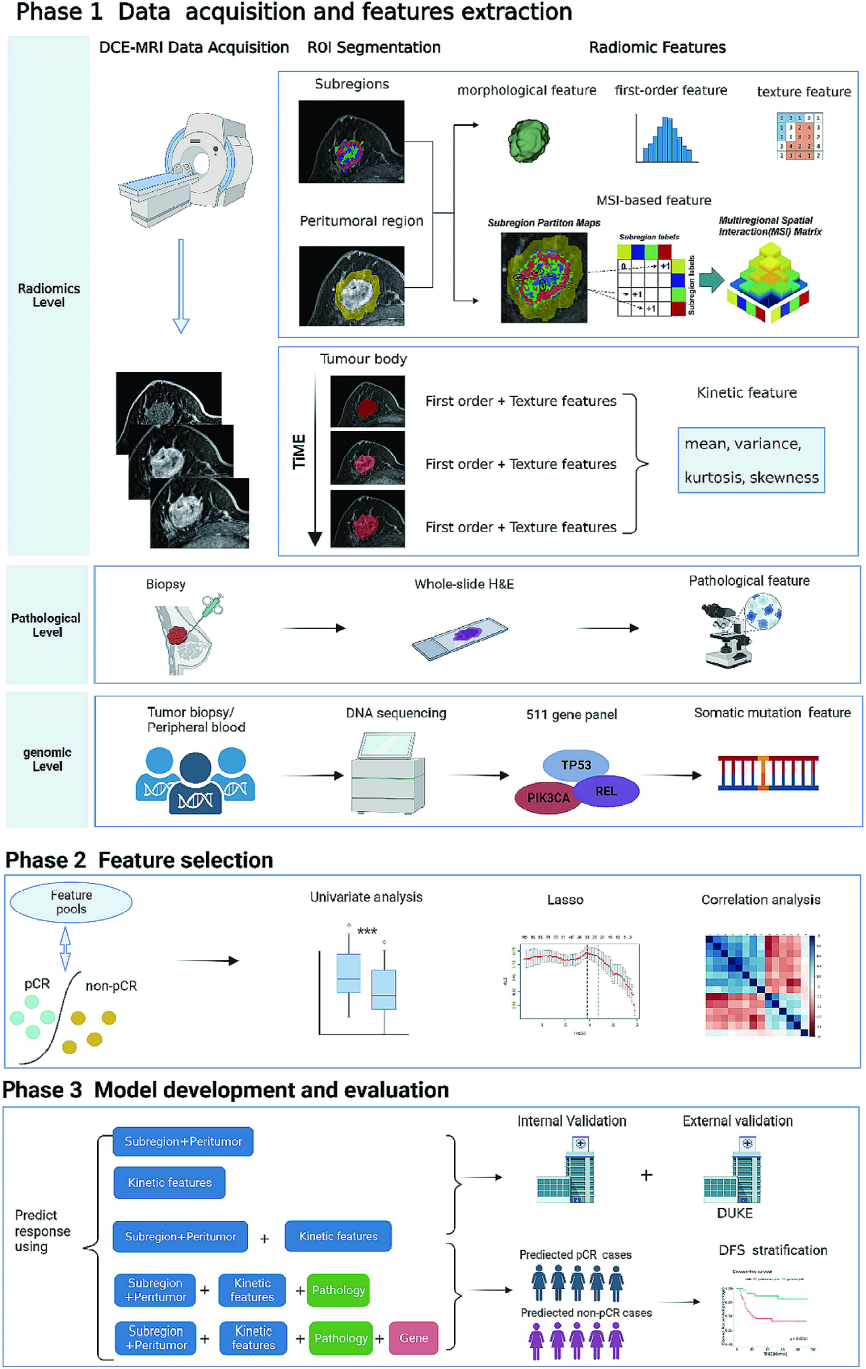
Overview of the study design Phase 1: DCE-MRI, clinicopathological and genetic data were collected, and radiomic, clinicopathological, and genomic features were extracted before treatment. Phase 2: Baseline individual radiomic, clinicopathological, and genomic features significantly associated with pCR were identified. Phase 3: The selected features were gradually integrated into the machine learning model, and the performance of the models for predicting pCR and prognosis in the internal and external validation sets was assessed. DCE-MRI = dynamic contrast material-enhanced magnetic resonance imaging; NAC = neoadjuvant chemotherapy; pCR = pathological complete response

We attempted to develop radiomic and radiogenomic models to simultaneously predict the NAC response and long-term prognosis of TNBC patients. In phase 1, we collected pretreatment DCE-MRI, clinicopathological, and DNA sequencing data. Radiomic features were extracted from the tumor body, subregions and peritumoral region. In phase 2, we identified the radiomic, clinicopathological, and genomic features significantly associated with pCR. In phase 3, we continuously integrated the selected features into the machine learning model to predict pCR. Three radiomics models were developed as follows: Model 1 (comprising subregional and peritumoral features), Model 2 (comprising kinetic features), and Model 3 (integrating the features of Model 1 and Model 2). The three models were tested in the internal and external validation sets. Then, pathological features were integrated to develop a pathology-radiomics model (PRM), and genomic features were further integrated to develop a genomics-pathology-radiomics model (GPRM). Finally, the prognostic implications of the models were assessed by measuring disease-free survival (DFS) using Kaplan‒Meier curves and multivariate Cox regression. The study procedure is shown in Fig. 2.

### Clinical, pathological, and prognostic data

The clinicopathological and prognostic data of all patients were collected. The clinical information included age, menopausal status, pre-NAC T stage and N stage, surgery type and NAC regimen. Pathological information included the pathological type, Ki-67 index and lymphovascular invasion before NAC and the pCR status after NAC. pCR was defined as the absence of invasive cancer burden in either the breast or associated axillary lymph nodes (ypT0/is ypN0). For prognostic information, we collected the date of progression (local recurrence and distant metastasis) to determine the duration (months) of DFS. DFS was calculated from the date of surgery to the date of progression, the last confirmation of no evidence of disease, or the most recent follow-up examination.

### DCE-MRI data

#### MRI technique and image preprocessing

At our center, scanning was performed on three types of scanners: a Siemens 3.0-T MRI scanner (Siemens Healthineers, Erlangen, Germany), an Aurora 1.5-T MRI scanner (Aurora Imaging Technology, Aurora Systems, Inc., Canada) and a GE 1.5-T MRI scanner (GE, Signa HDx) with a 16-channel body coil. In the DUKE dataset, scanning was performed using a 1.5-T or 3-T breast DCE-MRI scanner. In the I-SPY 1 dataset, scanning was performed using a 1.5-T breast DCE-MRI scanner. All patients were scanned in the prone position. The detailed scanning parameters and image preprocessing procedure are presented in Additional file 2.

### Region of interest (ROI) segmentation

#### Segmentation of the tumor body and peritumoral region

Tumor body segmentation was performed manually by two radiologists with more than ten years of experience using ITK-SNAP (version 3.8.0). The 3D segmentation ROIs of the tumor body were first delineated in the early postcontrast phase of DCE-MRI and then propagated to the precontrast and late postcontrast phases. The peritumoral region was obtained by expanding the tumor outward to a width of 5 mm and subtracting the tumor region [30, 31]. Intraclass correlation coefficients (ICCs) were utilized to evaluate the intra- and interobserver agreement in terms of feature extraction. The radiologists were blinded to the clinicopathological information.

#### Segmentation of the intratumoral subregions

**Fig. 3:**
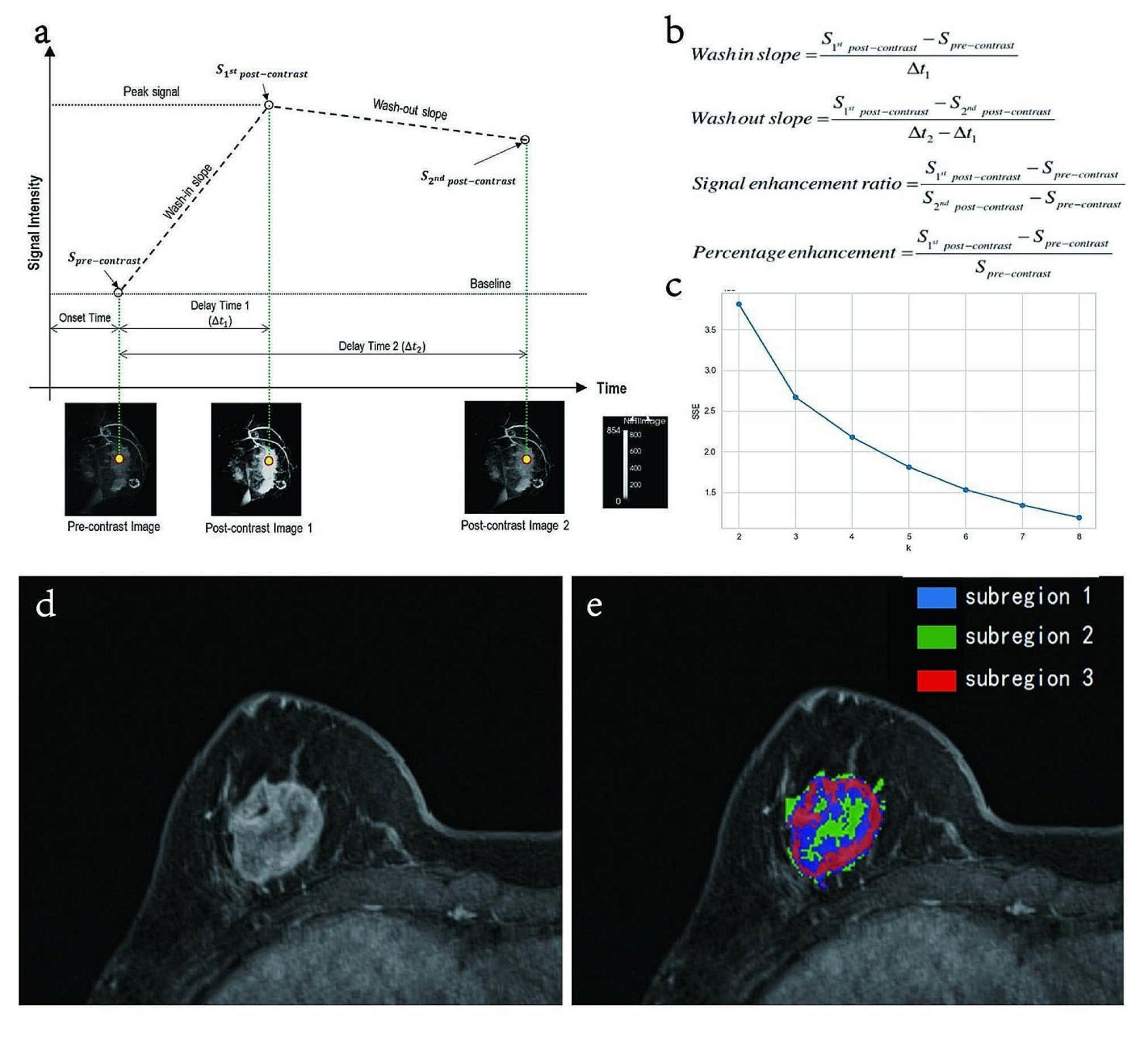
Illustration of subregion segmentation. (**a/b**) Calculation method for four kinetic parameters. (**c**) K-means clustering was used to obtain the optimal clustering centers. (**d**) Original image of a breast tumor. (**e**) Outcome of subregion segmentation of the breast tumor. The breast tumor was divided into three subregions. Subregions 1, 2, and 3 represent the poorly, moderately, and highly perfused subregions, respectively

We referred to Wu et al.‘s article to segment each tumor into multiple phenotypically consistent subregions based on four kinetic parameters of DCE-MRI [32]. First, the pixel values of the same pixel in different periods of enhancement were extracted and transformed into feature vectors, through which the four kinetic parameters of each pixel were calculated, including the wash-in slope (WIS), wash-out slope (WOS), signal enhancement ratio (SER) and percentage enhancement (PE). The algorithm is shown in Fig. 3a, b. Then, these feature vectors were clustered by the unsupervised k-means algorithm, and the best results were achieved when the number of clusters was 3 (Fig. 3c). In three different clusters (subregions), each of the four kinetic parameters increased from subregion 1 to subregion 3. We thus considered subregions 1, 2, and 3 to represent the poorly, moderately, and highly perfused subregions of the tumor, respectively (Fig. 3d, e).

### Radiomics feature extraction

#### Radiomics and MSI-based features from subregions and peritumoral regions

Based on the multiregional maps, we analyzed the characteristics of each region separately and the correlations among them. We extracted 1414 radiomic features, including morphological features, first-order features, texture features and features processed by filters from each intratumoral subregion and the peritumoral region. We used the multiregional spatial interaction (MSI) matrix to characterize and quantify spatial heterogeneity [32]. Then, we extracted 22 features from the MSI matrix, including 18 first-order and 4 s-order features.

#### Kinetic features from the tumor body

To reflect the hemodynamic heterogeneity of TNBC patients, we investigated the potential value of the variation in image texture over time. We extracted kinetic features of the tumor body, including the mean, variance, kurtosis and skewness of the phase-varying curve constructed based on feature values in all phases, for each first-order and textural feature. The process of feature extraction is shown in Fig. 2, and more details are shown in Additional file 3.

### Radiomics feature selection

Before further analysis, all the extracted radiomic features were standardized with z scores to eliminate the differences in the value scales of the data. To balance the dataset, a synthetic minority oversampling technique (SMOTE) was employed to resample the training set.

The ICCs between the features extracted from the ROIs delineated by the two radiologists were calculated, and the features with ICCs < 0.75 were eliminated. The remaining features were tested by univariate analysis, and the features with significant differences between pCR patients and nonpCR patients were selected. The Pearson correlation coefficients (PCCs) were calculated between features. When the coefficient was > 0.9, one of the features was randomly eliminated. Finally, we used the least absolute shrinkage and selection operator (LASSO) algorithm combined with 10-fold cross-validation to screen the top radiomic features derived from the intratumoral subregions and peritumoral regions. Using the same method, the top radiomic features from the kinetic features were screened out. These two selected sets of radiomic features constituted the feature subsets of the radiomic models.

Logistic regression was used for multivariate analysis of the selected top radiomic features, and the independent risk factors were used to develop radiomic models to predict pCR. Multicollinearity was evaluated by the variance inflation factors (VIFs) for variables in the model. Variables with VIFs > 10 indicated severe multicollinearity [33].

### Genomics data

Fresh tumor tissues obtained using baseline core-needle biopsy and matched white blood cell samples were collected, and genomic DNA was sequenced using the 511-gene panel. The 511-gene panel comprises 511 genes closely related to the development and targeted therapy of breast cancer in The Cancer Genome Atlas (TCGA) database and other databases. Based on second-generation sequencing technology, the exons and partial introns of the 511 genes were enriched by hybridization with a biotin probe. DNA sequencing provides targeted and in-depth detection of high-risk genes to accurately detect gene mutations, copy number variations and other events that have definite clinical relevance to breast cancer. Genomic DNA from both tissue samples and matched white blood cell samples was sequenced to distinguish somatic mutations from germline mutations. This study focused exclusively on somatic genomic alterations. The specific details of sample preparation and sequencing data generation can be found in Additional file 4 and our previous work [29, 34].

We saved the DNA sequencing results as ‘fastq’ files. We analyzed the sequencing results using the algorithm for gene mutation acquisition published by Broad and obtained the gene mutation results and annotated them. The main steps include quality control of the ‘fastq’ file, genomic mating, analysis of somatic and germline mutations, and annotation. We saved somatic mutations in mutation annotation format (MAF). The mutation data were summarized, analyzed, annotated, and visualized using Maftools in R version 4.2.2.

The mutation information analyzed included mutation status, mutation frequency and variant allele frequency (VAF). Mutation status refers to whether the CDS region of a gene has a nucleotide mutation that can cause a change in the encoding amino acid (nonsynonymous mutations). Mutation frequency refers to the total number of nonsynonymous mutations occurring in the CDS region of a gene. VAF refers to the percentage of mutant alleles at a specific locus. We summed the VAF values of the nonsynonymous mutation sites in each gene. We used the z score to standardize these three types of features to reduce interference during model development. Pearson’s chi-square test was employed to compare unordered categorical variables. A t test was used to identify VAF features that were significantly different between pCR patients and nonpCR patients.

### Development of radiomic and radiogenomic models

We used the radiomic features from two feature subsets to develop three radiomic models by logistic regression. Subregional and peritumoral features were used to develop Model 1, and kinetic features were used to develop Model 2. Finally, Model 3 was developed by integrating the features of Model 1 and Model 2. These models were validated with internal and external validation sets.

For clinicopathological and genomics features, univariate analysis was used to select features that were significantly different between pCR patients and nonpCR patients. The selected features were combined with the radiomics score (Radscore) of the optimal radiomics model to develop a pathology-radiomics model (PRM) and a genomics-pathology-radiomics model (GPRM) using logistic regression.

### Performance of the models for predicting NAC response and prognosis

The performance of these models for predicting pCR was evaluated by the area under the curve (AUC), accuracy, sensitivity and specificity. The DeLong test was applied to compare the AUC values between different models [35].

The prognostic implications of the optimal radiomic model, PRM and GPRM were assessed in the training and validation sets. The cutoff value was calculated with pCR as the endpoint, and the patients were divided into predicted pCR and predicted nonpCR groups. Kaplan‒Meier curves were used to assess whether the model could stratify patients by DFS. For PRM and GPRM, multivariate Cox proportional hazards regression was used to investigate whether the pCR predicted by the radiomic model added independent information in the presence of the covariates of pathological and genomic features.

### Statistical analysis

The data analyses and processes were implemented with Python (version 3.6) and R software (version 4.2.2). Continuous variables were summarized as the mean ± SD, and categorical variables were described as the number of patients and percentage. Continuous variables were compared by two-sample t tests, while qualitative variables were analyzed by the chi-square test or Fisher’s exact test. For all tests, *P* < 0.05 was considered to indicate statistical significance.

For the radiomic features, the ICCs were used to evaluate the consistency of the radiomic features extracted from the ROIs delineated by two different radiologists, and an ICC ≥ 0.75 was considered to indicate high consistency. Univariate analysis, correlation analysis, LASSO regression, and logistic regression were used to select key features to predict pCR. Receiver operating characteristic (ROC) curves were used to evaluate the different models, and the AUC with 95% confidence intervals (CIs), accuracy, sensitivity, and specificity were calculated. The DeLong test was performed to compare the AUCs of the different models, and *P* < 0.05 was considered to indicate statistical significance. Kaplan-Meier curves and multivariate Cox proportional hazards regression were used to assess the prognostic implications of the models.

## Results

### Patient characteristics

A total of 315 patients were initially treated at our hospital. All patients received 4 or 8 cycles of NAC treatment. The regimens were based on either taxane or taxane combined with anthracycline. The pCR rate of these patients was 39.7% (125/315), and the nonpCR rate was 60.3% (190/315). Fifty-three patients (16.8%) underwent breast-conserving surgery, and 262 patients (83.2%) underwent mastectomy. The median follow-up time was 34 months (range, 1–93 months). In the DUKE cohort (*n* = 50), the pCR rate was 38.0% (19/50), and the nonpCR rate was 62.0% (31/50). The mean age of the patients was 50.24 years (25.01–73.32). There were 8 (16%) patients with stage T greater than 2, and 11 (22%) patients with stage N greater than 1. In the I-SPY 1 cohort (*n* = 27), the pCR rate was 44.4% (12/27), and the nonpCR rate was 55.6% (15/27). The mean age of the patients was 47.34 years (33.47–68.31).

In the radiomics development cohort, there were 223 patients in the training set and 92 patients in the validation set. The baseline clinicopathological characteristics of the patients in the pCR and nonpCR groups in the training set are shown in Table 1. Ki-67 and lymphovascular invasion were significantly different between the pCR and nonpCR patients (*P* = 0.041 and 0.001, respectively). In the radiogenomic development cohort, there were 69 patients in the training set and 29 patients in the validation set. The VAFs of REL and MED23 were significantly different in the training set (*P* = 0.018 and 0.025, respectively). Mutations in MED23 and REL were more common in the nonpCR patients. More information is summarized in Additional file 5.

**Table 1:** Clinicopathological characteristics of patients in the pCR and nonpCR groups in the training set

Characteristic	pCR (*n* = 90)		NonpCR (*n* = 133)		*P* Value
Age (y)	N	%	N	%	0.447
Mean ± SD	47.31 ± 11.51		48.50 ± 11.45		
Menopausal status					0.554
Menopausal	45	50.0	72	54.1	
Premenopausal	45	50.0	61	45.9	
Ki-67 status					0.041*
< 20%	0	0	6	4.5	
≥20%	90	100.0	127	95.5	
Surgery type					0.569
Breast conservation	19	21.1	24	18.0	
Mastectomy	71	78.9	109	82.0	
T stage					0.223
1	14	15.6	14	10.5	
2	57	63.3	80	60.2	
3	9	10.0	26	19.5	
4	10	11.1	13	9.8	
N stage					0.917
0	18	20.0	30	22.6	
1	45	50.0	64	48.1	
2	15	16.7	19	14.3	
3	12	13.3	20	15.0	
Lymphovascular invasion					< 0.001*
Present	1	1.1	61	45.9	
Absent	89	98.9	72	54.1	
Pathological type					0.063
IDC	90	100.0	128	96.2	
ILC, IMPC	0	0	5	3.8	

IDC = invasive ductal carcinoma; ILC = invasive lobular carcinoma; IMPC = invasive micropapillary carcinoma. *, *P* < 0.05

### Performance of the radiomic models for predicting pCR

In total, 11,258 radiomic features were extracted. After feature selection, 5 radiomic features from subregions and 2 radiomic features from the peritumoral region were included in Model 1. Eighteen kinetic features from the tumor body were included in Model 2. Model 3 integrated 25 features of Model 1 and Model 2. The detailed process is summarized in Additional file 6.

In the validation set, both Model 1 (AUC = 0.74) and Model 2 (AUC = 0.73) could effectively predict pCR. The predictive accuracy of Model 3 improved (AUC = 0.79). In the external validation set, Model 3 (AUC = 0.78) also performed better than Model 1 (AUC = 0.73) and Model 2 (AUC = 0.66). The VIFs in these models were all less than 10, indicating that there was no multicollinearity among these variables.

### Improved performance of radiogenomic models for predicting pCR

In the training set of the radiomics development cohort, a fusion model (PRM) was developed by integrating Ki-67 expression and lymphovascular invasion with the Radscore of Model 3 to predict pCR. In the validation set, the AUC (0.88 vs. 0.79, *P* = 0.003) and specificity (0.74 vs. 0.53) of the PRM were greater than those of Model 3.

In the training set of the radiogenomic development cohort, the VAFs of the REL and MED23 were further integrated into the PRM to develop a radiogenomic model (GPRM). In the validation set, with the integration of features, the AUCs of Model 3, the PRM and the GPRM improved continuously and were 0.75, 0.86 and 0.93, respectively. The DeLong test showed that the GPRM further improved the performance for predicting pCR compared with Model 3 (AUC: 0.75 vs. 0.93; *P* = 0.019). The specificities of Model 3, the PRM and the GPRM were 0.65, 0.83 and 0.91, respectively. In the validation sets of the radiomics and radiogenomics development cohorts, as the features of the model continued to be integrated, the net clinical benefit for patients continued to improve.

**Fig. 4:**
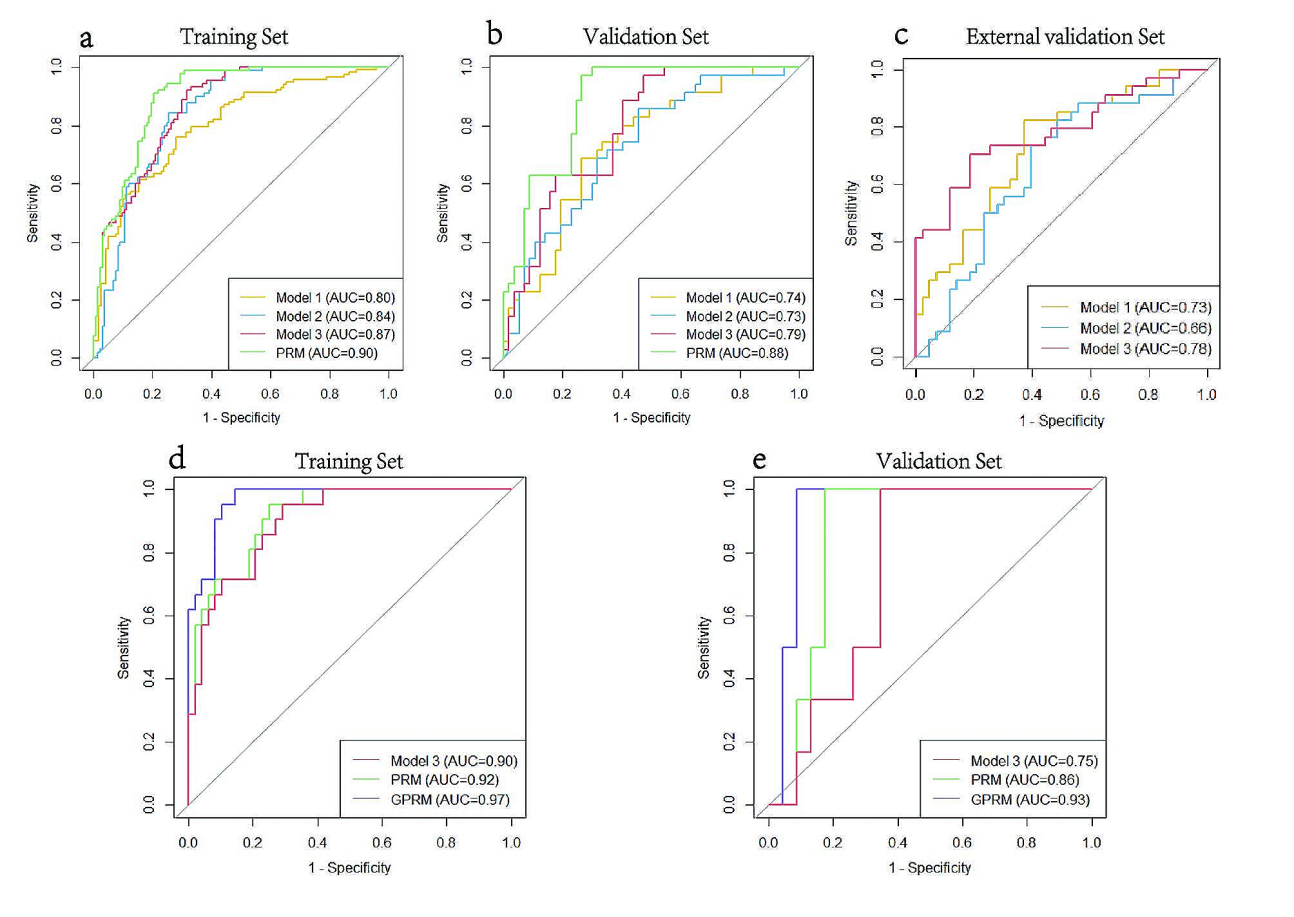
Predictive performances of the different models **(a-e).** Plots show the receiver operating characteristic (ROC) curves of the different models in the training set (**a**) and validation set (**b**) of the radiomics development cohort. The plot shows the ROC curves of the different models in the external validation set (**c**). The plot shows the ROC curves of different models in the training set (**d**) and validation set (**e**) of the radiogenomic development cohort. Model 1, radiomics model constructed by features from the subregions and peritumoral region; Model 2, radiomics model constructed by kinetic features from the tumor body; Model 3, radiomics model integrating features of Model 1 and Model 2; PRM = pathology-radiomics model; GPRM = genomics-pathology-radiomics model

**Fig. 5:**
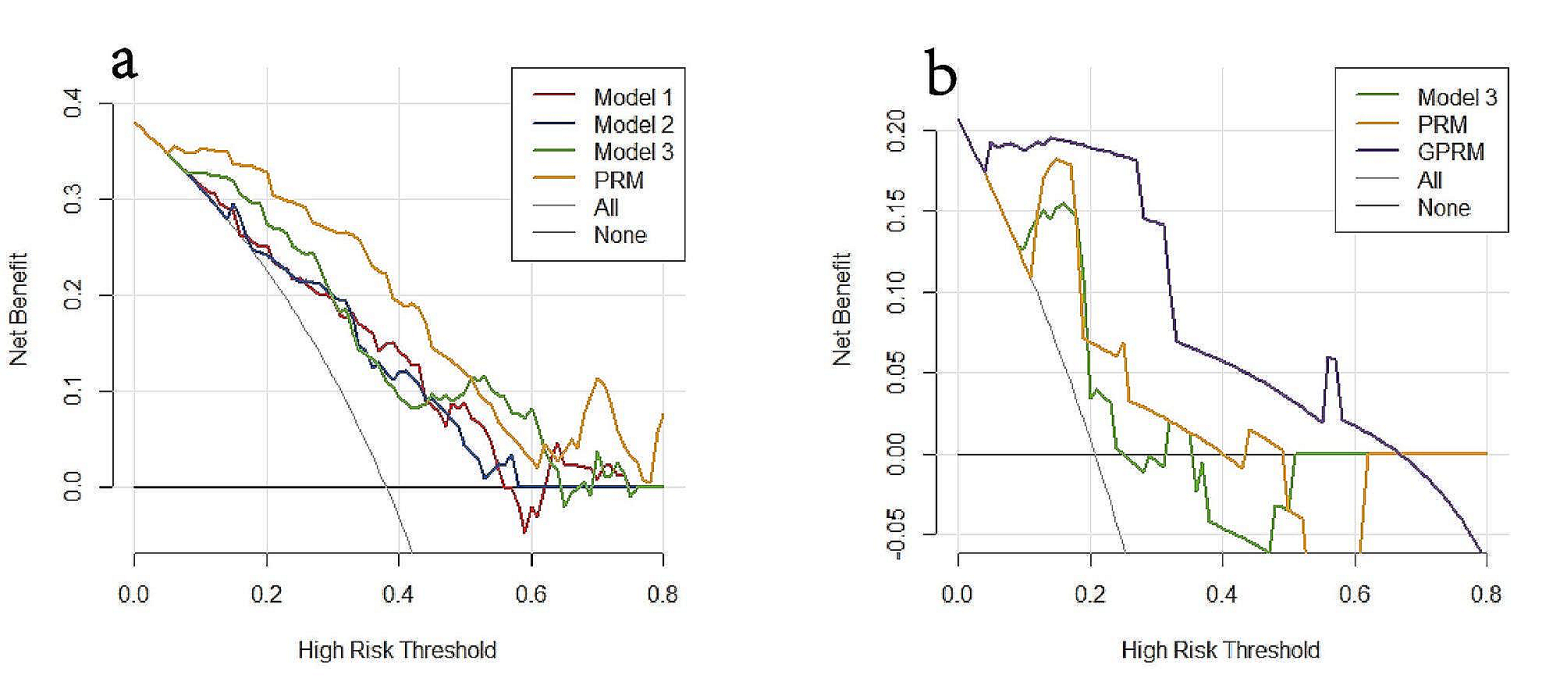
Decision curves of different models in the validation sets of the radiomic development cohort (**a**) and the radiogenomic development cohort (**b**). Model 1, radiomics model constructed by features from the subregions and peritumoral region; Model 2, radiomics model constructed by kinetic features from the tumor body; Model 3, radiomics model integrating features of Model 1 and Model 2; PRM = pathology-radiomics model; GPRM = genomics-pathology-radiomics model

In summary, we constructed three radiomic models and two fusion models. Tables 2 and 3 show the performance of these models and the P values from the ROC analysis. Figures 4 and 5 show the receiver operating characteristic (ROC) curves and the decision curves generated by different models. The specific formulas of these models are shown in Additional file 7.

**Table 2:** Performance of the predictive models in the training set, validation set and external validation set

Cohort	Model	Training set				Validation set				External validation set			
		AUC(95% CI)	Accuracy	Sensitivity	Specificity	AUC(95% CI)	Accuracy	Sensitivity	Specificity	AUC(95% CI)	Accuracy	Sensitivity	Specificity
Radiomics development cohort	Model 1	0.80(0.75–0.86)	0.74	0.76	0.72	0.74(0.63–0.84)	0.72	0.69	0.74	0.73(0.62–0.84)	0.71	0.64	0.82
	Model 2	0.84(0.79–0.89)	0.78	0.83	0.74	0.73(0.63–0.83)	0.66	0.86	0.54	0.66(0.54–0.78)	0.66	0.60	0.74
	Model 3	0.87(0.82–0.91)	0.78	0.92	0.68	0.79(0.70–0.88)	0.70	0.97	0.53	0.78(0.67–0.89)	0.81	0.83	0.79
	PRM	0.90(0.86–0.94)	0.84	0.91	0.80	0.88(0.81–0.95)	0.83	0.97	0.74	-	-	-	-
Radiogenomics development cohort	Model 3	0.90(0.83–0.97)	0.78	0.95	0.71	0.75(0.57–0.93)	0.72	1.00	0.65	-	-	-	-
	PRM	0.92(0.85–0.98)	0.81	0.95	0.75	0.86(0.73–0.99)	0.83	0.83	0.83	-	-	-	-
	GPRM	0.97(0.94-1.00)	0.90	1.00	0.85	0.93(0.84-1.00)	0.93	1.00	0.91	-	-	-	-

Model 1 was a radiomic model constructed by features from subregions and the peritumoral region; Model 2 was a radiomic model constructed by kinetic features from the tumor body; Model 3 was a radiomic model integrating features from Model 1 and Model 2; PRM = pathology-radiomics model; GPRM = genomics-pathology-radiomics model; AUC = area under the receiver operating characteristic (ROC) curve

**Table 3:** The P value of the Delong test on the training set, validation set and external validation set of the predictive models

Cohort	Model	Training set				Validation set				External validation set	
		Model 1	Model 2	Model 3	PRM	Model 1	Model 2	Model 3	PRM	Model 1	Model 2
Radiomics development cohort	Model 2	0.047*	-	-	-	0.873	-	-	-	0.436	-
	Model 3	0.009*	0.302	-	-	0.196	0.076	-	-	0.464	0.101
	PRM	< 0.001*	0.009*	0.006*	-	0.003*	< 0.001*	0.003*	-	-	-
Radiogenomics development cohort	PRM	-	-	0.218	-	-	-	0.066		-	-
	GPRM	-	-	0.015*	0.030	-	-	0.019*	0.163	-	-

Model 1 was a radiomics model constructed by features from the subregions and peritumoral region; Model 2 was a radiomics model constructed by kinetic features from the tumor body; Model 3 was a radiomics model integrating features of Model 1 and Model 2; PRM = pathology-radiomics model; GPRM = genomics-pathology-radiomics model; *, *P* < 0.05

### Assessment of the prognostic implications of the models

**Fig. 6:**
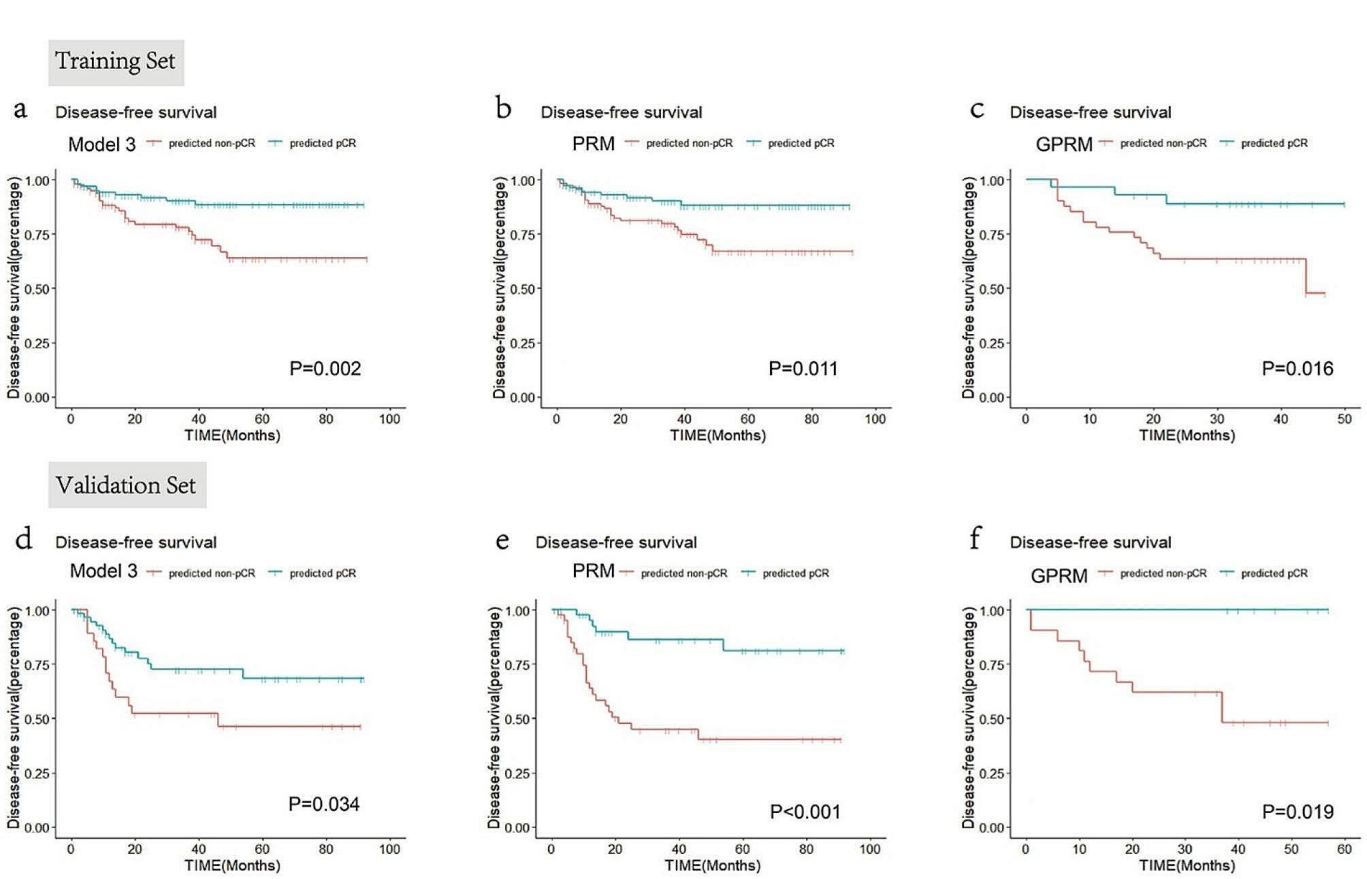
Kaplan‒Meier survival analyses according to the predicted pCR status generated by the three models for disease-free survival. Model 3, radiomics model integrating features of Model 1 and Model 2; PRM = pathology-radiomics model; GPRM = genomics-pathology-radiomics model

The optimal cutoff values generated by the ROC curves of Model 3, the PRM and the GPRM were 0.31, 0.37 and 0.16, respectively. Using these threshold values, patients were classified into a predicted pCR group and a predicted nonpCR group. As shown in Fig. 6, Kaplan‒Meier curves showed that the predicted pCR group had better DFS in the training set (*P* = 0.002, 0.011 and 0.016, respectively) and validation set (*P* = 0.034, 0.001 and 0.019, respectively).

As shown in Table 4, in the multivariate Cox analysis, for the variables in the PRM and GPRM, pCR predicted by Model 3 remained independently associated with DFS after we adjusted for pathological and genomic risk factors (PRM: DFS odds ratio, 0.168, 95% CI: 0.045–0.626, *P* = 0.008; GPRM: DFS odds ratio, 0.171; 95% CI: 0.029–1.019; *P* = 0.052).

**Table 4:** Cox multivariate analysis of the associations of variables in the PRM and GPRM with disease-free survival

Variable name	Multivariate analysis of PRM			Multivariate analysis of GPRM		
	OR	95% CI	*P* value	OR	95% CI	*P* value
Radscore of Model 3	0.168	0.045–0.626	0.008*	0.171	0.029–1.019	0.052
Ki-67 status	0.943	0.126–7.031	0.954	0.885	0.112-7.000	0.908
Lymphovascular invasion	0.719	0.353–1.464	0.363	0.598	0.236–1.513	0.278
VAF of REL	-	-	-	0	0	0.446
VAF of MED23	-	-	-	0	0	0.301

PRM, pathology-radiomics model; GPRM, genomics-pathology-radiomics model; VAF, variant allele frequency; OR, odds ratio; CI, confidence interval. *, *P* < 0.05

## Discussion

To achieve individualized precision treatment, we developed a more accurate model to predict both pCR and prognosis in TNBC patients. The AUCs of the multiregional radiomic model (Model 3) were 0.79 and 0.78 in the internal and external validation sets, respectively. The radiogenomic model comprising pathological features (GPRM) could predict pCR more accurately, with an AUC of 0.93 in the validation set. Moreover, both radiomic and radiogenomic models could predict recurrence and metastasis.

Spatial heterogeneity has a significant impact on treatment response and patient prognosis [36, 37]. Wu et al. used subregional analysis to characterize intratumoral spatial heterogeneity [32], and Shi et al. used quadratic clustering to further promote the development of subregional correlation precision imaging [38]. Shi et al. and Wu et al. reported that intratumoral spatial heterogeneity was associated with pCR and prognosis, respectively [32, 38]. We partitioned the tumors into multiple spatially segregated, phenotypically consistent subregions. We analyzed the radiomic features of each subregion separately and their interrelationships to clearly show spatial heterogeneity. Compared with the studies of Wu et al. [32], in addition to the 22 MSI-based features, our study extracted radiomics features from each subregion and discovered that radiomics features had greater predictive value during the feature screening process. We combined intratumoral and peritumoral features to avoid missing the added value of the tumor microenvironment. In addition, we accounted for the image texture changes over enhancement time to characterize hemodynamic heterogeneity. We found that multiscale heterogeneity characterized by baseline multiregional quantitative radiomic features could robustly predict the NAC response.

The addition of genomic data to the model facilitates the discovery of new biomarkers to enhance predictive value [29, 39]. However, few studies have integrated multiomics to develop models, possibly due to the risk of invasive biopsies and the complexity of multidimensional data analysis. Meanwhile, it is meaningful to synthesize multidimensional information such as radiomic, pathological, and genomic features to describe tumor characteristics more comprehensively and develop more robust models. Stephen-John et al. collected clinical, digital pathological, genomic and transcriptomic features of breast cancer and found that the fusion model showed the highest performance for predicting pCR (AUC = 0.87) [39]. We found that mutations in MED23 and REL were more common in nonpCR patients. Our team’s previous finding that the MED23 p.P394h mutation could induce epirubicin resistance by affecting homologous recombination repair may provide an explanation [29]. Compared to the radiomics model, the fusion model exhibited a significant improvement in the AUC and specificity. In both the training set and the validation set, the AUC of the GPRM was significantly greater than that of Model 3, with P values of 0.015 and 0.019, respectively. This facilitates the identification of patients in whom pCR may not be achieved and the need for early adjustment of treatment, such as in combination with immunotherapy [40, 41] or bevacizumab [42], to increase the likelihood of achieving pCR and ultimately improve prognosis.

A multitask model that can predict both response and prognosis could better guide clinical decision making. Fan et al. reported that a predictive model for the Oncotype DX recurrence score was useful for both predicting pCR and prognosis in patients with breast cancer [43]. This approach is similar to transfer learning in principle, where trained markers are transferred to enhance the prediction accuracy for different clinical tasks. We found that specific features for predicting pCR were also effective for stratifying patients according to DFS. Moreover, the pCR predicted by the radiomic model had independent prognostic value and was positively correlated with good DFS in PRM. Possibly due to the small sample size, pCR predicted by Model 3 was positively associated with a good prognosis in GPRM but was not statistically significant. Increasing the sample size may improve the statistical power of radiomic features. Our multitask model predicts the pCR and prognosis of TNBC patients simultaneously, helping to identify patients for whom pCR may not be achieved to facilitate the realization of individualized treatment.

Our study had several limitations. First, our radiogenomic model should be further tested in independent, larger cohorts. Second, it would be of interest to combine DCE-MRI with other imaging modalities, such as diffusion-weighted MR imaging, to further improve the prediction accuracy. Third, it would also be worthwhile to increase the interpretability of our models and identify new meaningful gene therapeutic targets to improve the prognosis of TNBC patients in future studies.

Our study focused on clinically used diagnostic DCE MR imaging and revealed that combining the radiomic features of multiple tumor regions facilitates the prediction of pCR and DFS. In addition, the integration of radiomic features with clinicopathological and genomic features could improve the prediction efficiency. We envision that the proposed methodology for defining and characterizing intratumoral spatial heterogeneity will be applicable to other cancers with similar poor prognoses. In future studies, it may be of interest to combine imaging with pathologic or molecular data to understand the underlying biological basis of the tumor heterogeneity captured by multiregional imaging features.

## Conclusion

Imaging multiscale heterogeneity could be used to predict the pCR of TNBC patients and advance tailored treatment in wider regions and populations. The radiogenomic model based on quantitative heterogeneity could serve as a valuable clinical marker to predict the pCR and prognosis of TNBC patients.

### Electronic supplementary material

Below is the link to the electronic supplementary material.

Supplementary Material 1

## Data Availability

The datasets used and/or analyzed during the current study are available from the corresponding author upon reasonable request. DUKE URL: 10.7937/TCIA.e3sv-re93. SPY 1 URL: 10.7937/K9/TCIA.2016.HdHpgJLK.

## Abbreviations

TNBC: Triple-negative breast cancer
NAC: Neoadjuvant chemotherapy
pCR: Pathological complete response
DCE-MRI: Dynamic contrast material-enhanced magnetic resonance imaging
PRM: Pathology-radiomics model
GPRM: Genomics-Pathology-Radiomics Model
DFS: Disease-free survival
ROI: Region of interest
WIS: Wash-in slope
WOS: Wash-out slope
SER: Signal enhancement ratio
PE: Percentage enhancement
MSI: Multiregion spatial interaction
ICC: Intraclass correlation coefficient
PCC: Pearson correlation coefficient
LASSO: Least absolute shrinkage and selection operator
MAF: Mutation annotation format
VAF: Variant allele frequency Radscore Radiomics score
AUC: Area under the curve
ROC: Receiver operating characteristic
CI: Confidence interval
