# Supplementary material for: A preoperative radiogenomic model based on quantitative heterogeneity for predicting outcomes in triple-negative breast cancer patients who underwent neoadjuvant chemotherapy
Source: Cancer Imaging. 2024 Jul 30;24:98. doi: 10.1186/s40644-024-00746-z (PMC11289960; doi:10.1186/s40644-024-00746-z)
Supplement: Supplementary file 1 — Supplementary Material 1 [file 40644_2024_746_MOESM1_ESM.docx]

**ELECTRONIC SUPPLEMENTARY MATERIAL**

**Additional file 1**

**Patients**

For patients from the DUKE dataset:

The inclusion criterion was (1) triple-negative breast cancer confirmed by a core-needle biopsy before treatment. The exclusion criteria were patients who had (1) no visible lesions, (2) poor quality MR images, (3) an incomplete MR sequence, or who did not receive neoadjuvant chemotherapy (NAC).

For patients from the I-SPY 1 dataset:

The inclusion criterion was (1) triple-negative breast cancer confirmed by a core-needle biopsy before treatment. The exclusion criteria were as follows: (1) patients with no visible lesions, (2) patients with poor-quality MR images, and (3) patients with incomplete MR sequences.

**Additional file E2**

**DCE-MRI data**

1. **MRI technique and image preprocessing**
   1. MRI technique

For the Siemens system, 3.0-T magnetic resonance was used, and the breast coil was a 16-channel phased array. The sequences included diffuse-weighted imaging (b= 1000 s/mm2, repetition time (TR)/echo time (TE) = 5600/75 ms, flip angle= 90°, field of view (FOV) = 180 × 300 mm^2^, slice thickness= 5 mm, slice gap= 0, band width=1666 Hz, EPI= 96) and dynamic contrast-enhanced sequences (TR/TE= 5.1/1.7 ms, flip angle= 15°, FOV = 260 × 260 mm^2^, slice thickness= 3 mm, slice gap= 0). The contrast agent Gd-DTPA (0.1 mmol/kg) was infused at a flow rate of 3.0 mL/s, followed by flushing with 15 mL of saline. Images from the precontrast phase and five contrast phases were collected. The acquisition time was 220 s.

For the Aurora system, 1.5-T magnetic resonance and a special breast coil were used. The sequences included axial T2-weighted turbo spin‒echo sequences with fat suppression (T2-weighted imaging, T2WI; TR= 6680 ms, TE= 68 ms, slice thickness = 3 mm, slice gap = 1 mm), T1-weighted gradient echo (T1-weighted imaging, T1WI; TR= 5 ms, TE= 13 ms, slice thickness = 3 mm, slice gap = 1 mm), and dynamic contrast–enhanced T1-weighted sequences with fat plus water suppression sequences (TR= 5 ms, TE= 29 ms, slice thickness= 1.5 mm, slice gap= 0, FOV= 360×360 mm^2^). The contrast agent Gd-DTPA (0.2 mmol/kg) was infused 90 s into the precontrast phase at a flow rate of 2.0 mL/s, followed by flushing with 20 mL of saline. Images from the precontrast phase and three contrast phases were collected. The acquisition time was 270 s.

For the GE system, 1.5-T magnetic resonance was used, and the breast coil was a 16-channel phased array. The sequences included axial T2-weighted turbo spin‒echo sequences with fat suppression (T2-weighted imaging, T2WI, TR= 6680 ms, TE= 68 ms, slice thickness = 3 mm, slice gap = 1 mm), T1-weighted gradient echo (T1-weighted imaging, T1WI; TR= 5 ms, TE= 13 ms, slice thickness = 3 mm, slice gap = 1 mm), and dynamic contrast–enhanced T1-weighted sequences with fat plus water suppression sequences (TR= 6.5 ms, TE= 3.5 ms, flip angle= 10°, slice thickness= 3 mm, slice gap= 0, FOV 300×300 mm^2^). The contrast agent Gd-DTPA (0.2 mmol/kg) was infused 90 s into the precontrast phase at a flow rate of 2.0 mL/s, followed by flushing with 20 mL of saline. Images from the precontrast phase and four contrast phases were collected. The acquisition time was 125 s.

For the DUKE dataset, imaging was performed using a 1.5-T or 3-T breast DCE-MRI scanner. The slice thickness ranged from 0.9 to 2.5 mm, the repetition time ranged from 3.54 to 7.39 ms, the echo time ranged from 1.25 to 2.76 ms, the field of view ranged from 250 to 480 mm, and the flip angle ranged from 7 to 12°.

For the I-SPY 1 dataset, imaging was performed using a 1.5-T breast DCE-MRI scanner. The slice thickness was 2.5 mm or less, the repetition time was 20 ms or less, the echo time was 4.5 ms, the field of view ranged from 160 to 180 mm, and the flip angle was less than 45°.

- 1. Image preprocessing

During image data preprocessing, all other phases were coregistered into the first postcontrast phase of DCE-MRI through nonlinear registration using the symmetric normalization algorithm, which was performed using the ANT toolbox (version 2.3.5), to eliminate the spatial mismatches caused by motion artifacts[1]. A nonparametric nonuniformity normalization algorithm was applied for bias field correction[2]. Then, we used the no-new-UNet (nnUNet) network to automatically segment the breast region. Moreover, z score normalization in the image domain was performed.

For data from different MRI scanners, to handle the inconsistency of the temporal points of the postcontrast phases, we defined the precontrast, early postcontrast and late postcontrast phases based on the tumor-to-background (fat) relative enhancement ratio ($TFRE ratio$)[3-5]. Specifically, we first calculated the ratio of the mean voxel intensity of the tumor region to that of the fat region for each phase of DCE ($TF ratio$):

$${TF ratio}_{i}=\frac{mean({Tumor}_{i})}{mean({Fat}_{i})}, i=1\ldots N$$

$N$ represents the total number of phases of the DCE sequence, where $i=1$ represents the precontrast phase and $i=2\ldots N$ represents each postcontrast phase. Then, $TFRE ratio$ was obtained by comparing the $TF ratio$ of each enhancement phase with the $TF ratio$ of the precontrast phase:

$${TFRE ratio}_{j}=\frac{{TF ratio}_{j}}{{TF ratio}_{1}}, j=2\ldots N$$

$TFRE ratio$ tended to increase and then decrease after contrast agent injection. We designated the phase with the largest $TFRE ratio$ as the early postcontrast phase; the postpeak phase had a lower $TFRE ratio$ and a ratio that was no more than 10% higher than that of the following phase, which was designated the late postcontrast phase (if there were too few phases and the above conditions were not met, we directly selected the last phase as the late postcontrast phase)[6].

**Additional file 3**

**DCE-MRI data**

1. **Radiomics feature extraction**

1. Radiomics features (1414 in total)

- 1. Original image (112):
     1. The five features included the mean, maximum and minimum values of all pixels, the number of pixels of the tumor label and the volume of the tumor label.
     2. Fourteen morphological features
     3. Eighteen first-order features
     4. Seventy-five texture features:

1. 24 gray-level co-occurrence matrix (GLCM) features;
2. 14 gray-level dependence matrix (GLDM) features;
3. 16 gray-level run length matrix (GLRLM) features;
4. 16 gray-level size zone matrix (GLSZM) features;
5. 5 neighboring gray tone difference matrix (NGTDM) features.

1.2 Data enhancement was performed on the original image (1302):

1.2.1 Wavelet: All combinations using high-pass and low-pass filters in each of the three dimensions were produced (LLH, LHL, LHH, HLL, HLH, HHL, HHH, and LLL).

1.2.2 Square: Take the square of the original pixels and linearly scale them back to the original range.

1.2.3 SquareRoot: Take the square roots of the absolute image intensities and scale them back to the original range.

1.2.4 Logarithm: The logarithm of the absolute intensity + 1 was taken, and the values were scaled to the original range.

1.2.5 Exponential: Using e^ (absolute intensity) to obtain the exponential value of the intensity, the values were scaled to the original range.

1.2.6 Gradient: Returns the magnitude of the local gradient.

1.2.7 LocalBinaryPattern2D: A local binary pattern is used for each slice.

The eight wavelet transforms included LLH, LHL, LHH, HLL, HLH, HHL, HHH, and LLL, from which 18 first-order features and 75 texture features were extracted. The same 93 features were extracted from the above remaining 6 transforms. A total of 93*14 = 1302 features were extracted.

A total of 1414 radiomics features were extracted from the three subregions and peritumoral region. Finally, a total of 5656 radiomic features were obtained.

1. Multiregional spatial interaction (MSI)-based features (22 in total)

Referring to Wu et al.[7], the adjacent regions of each tumor voxel were probed, and the resulting pair was added to the corresponding entry in the MSI matrix, as shown in Figure S1. This process was repeated until all tumor voxels were iterated, and the spatial heterogeneity was summarized in the final MSI matrix. We included the peritumoral region as one distinct region to explicitly account for the spatial relationship between the tumor subregions and their surrounding tissue. A total of 22 features were extracted from the MSI matrix, including 18 first-order and 4 second-order statistical features, as shown in Table S1.

1. Kinetic features (5580 in total)

Kinetic features indicate the mean, variance, skewness and kurtosis of the enhancement phase-varying curves that represent the changes in features after the contrast agent was injected over time. Kinetic features were extracted based on each spatial domain feature except for the shape features. A total of 1395 first-order and texture features extracted from the precontrast, early postcontrast and late postcontrast phases of DCE-MR images of the tumor body were used to calculate kinetic features. Here, $dyn0$, $dyn1$ and $dyn2$ represent the precontrast phase, the early enhancement phase and the late enhanced phase, respectively.

- 1. Mean
  $mean({DYN=[dyn}_{1}\ldots{dyn}_{N}])=\frac{1}{N}\sum_{i=1}^{N} {dyn}_{i}$
  2. Variance
     $variance({DYN=[dyn}_{1}\ldots{dyn}_{N}])=\frac{1}{N-1}\sum_{i=1}^{N} {({dyn}_{i}-\bar{DYN})}^{2}$
  3. Skewness
     $skewness\left( {DYN=[dyn}_{1}\ldots{dyn}_{N}] \right)=\frac{1}{N}\sum_{i=1}^{N} \left[ \frac{{dyn}_{i}-\bar{DYN}}{\sigma} \right]^{3}$
  4. Kurtosis

$$kurtosis\left( {DYN=[dyn}_{1}\ldots{dyn}_{N}] \right)=\left\{ \frac{1}{N}\sum_{i=1}^{N} \left[ \frac{{dyn}_{i}-\bar{DYN}}{\sigma} \right]^{4} \right\}-3$$

By calculating the kinetic features separately for 1395 features, 1395*4=5580 kinetic features were obtained.

Finally, 11258 features were obtained, including 1414*4 radiomics features in three subregions and the peritumoral region, 22 MSI-based features and 5580 kinetic features.

**Additional file 4**

**Genomics data**

The 511-gene panel comprises 511 genes closely related to the development and targeted therapy of breast cancer in The Cancer Genome Atlas (TCGA) database and other databases. The 511-gene panel contains high-risk mutated genes associated with a high incidence of breast cancer, including 401 genes with mutations and 110 genes with copy number variations. The details are presented in Table S2.

**Additional file 5**

**Patient characteristics**

In the radiomics development cohort (n = 315), there were no significant differences in the baseline characteristics of patients between the training and validation sets (Table S3). The pCR rates of the training set and the validation set were 40.4% and 38.0%, respectively. There were 36 (16.1%) recurrences in the training set and 28 (30.4%) recurrences in the validation set; among these patients, 57 patients had distant metastasis (6 patients also had additional local-regional recurrence), and 7 patients had local-regional recurrence only.

In the radiogenomic development cohort (n = 98), 21 (30.4%) patients in the training set and 6 (20.7%) patients in the validation set achieved pCR. There were 21 (30.4%) recurrences in the training set and 11 (37.9%) recurrences in the validation set. For genomic features, 5880 somatic mutations were identified, including 5442 single nucleotide mutations and 438 insertion/deletion mutations. The median numbers of nonsynonymous single nucleotide mutations and insertion/deletion mutations were 45 and 4, respectively.

**Additional file 6**

**Radiomics feature selection**

We extracted a total of 11,258 radiomic features. First, to ensure the reproducibility of the features, 506 features with an ICC < 0.75 were excluded. The Wilcoxon signed-rank test showed that 677 features were significantly associated with pCR. Finally, LASSO regression was used to select the top features in the subregion + peritumoral feature pool and the kinetic feature pool. A total of 55 features were selected, namely, 11 subregional radiomic features, 6 peritumoral radiomic features and 38 kinetic features. For these 55 top features, multivariate logistic regression was performed to identify independent risk factors. Model 1 and Model 2 were established based on the results of multivariate logistic regression. Finally, 5 radiomic features were selected from the subregions, 2 radiomic features were selected from the peritumoral region, and 18 kinetic features were selected from the tumor body. The specific features included in each model are shown in Figure S2.

**Additional file 7**

**Radscore formula for Model 1, Model 2, Model 3, the PRM and the GPRM**

**Model 1-Radscore**= -0.762*subregion_1_original_Minimum+ -0.406*subregion_1_firstorder_Variance+ 0.822*subregion_2_gradient_glszm_LowGrayLevelZoneEmphasis+

-0.272*subregion_2_wavelet-LHH_glrlm_LongRunHighGrayLevelEmphasis+ 1.025*subregion_3_square_firstorder_Skewness+0.677*peritumor_wavelet-HHL_firstorder_Mean+

-0.463*peritumor_wavelet-LLL_glcm_MCC+ 0.098

**Model 2-Radscore**= -0.948*Skewness_original_gldm_SmallDependenceLowGrayLevelEmphasis+

-1.036*Skewness_exponential_glszm_ZoneVariance+ -0.698*Skewness_logarithm_firstorder_90Percentile+

-0.667*Skewness_logarithm_firstorder_Skewness+ -1.457*Skewness_squareroot_glcm_Imc2+

-1.181*Skewness_wavelet-HLH_gldm_SmallDependenceLowGrayLevelEmphasis+

1.000*Skewness_wavelet-LLL_glszm_LargeAreaHighGrayLevelEmphasis+ 4.795*Kurtosis_gradient_ngtdm_Coarseness+ 0.855*Kurtosis_Localbinarypattern2D_glcm_DifferenceEntropy+

-0.714*Kurtosis_Localbinarypattern2D_glrlm_RunEntropy+

0.387*Kurtosis_Localbinarypattern2D_glrlm_RunLengthNonUniformityNormalized+

-1.324*Kurtosis_wavelet-LLH_glcm_DifferenceEntropy+ -0.850*Kurtosis_wavelet-LHL_glcm_Imc1+

-1.994*Kurtosis_wavelet-LHL_gldm_DependenceEntropy+

-2.188*Kurtosis_wavelet-LHH_gldm_LargeDependenceEmphasis+

1.576*Kurtosis_wavelet-HLL_glrlm_ShortRunEmphasis+ 1.663*Kurtosis_wavelet-HLH_glcm_SumEntropy+

-0.354*Mean_wavelet-LHL_ngtdm_Complexity+ 0.217

**Model 3-Radscore**= -0.510*subregion_1_original_Minimum+ -0.312*subregion_1_firstorder_Variance+ 0.486*subregion_2_gradient_glszm_LowGrayLevelZoneEmphasis+

-0.507*subregion_2_wavelet-LHH_glrlm_LongRunHighGrayLevelEmphasis+ 0.692*subregion_3_square_firstorder_Skewness+0.366*peritumor_wavelet-HHL_firstorder_Mean+

-0.264*peritumor_wavelet-LLL_glcm_MCC+

-0.395*Skewness_original_gldm_SmallDependenceLowGrayLevelEmphasis+

-0.159*Skewness_exponential_glszm_ZoneVariance+ -0.354*Skewness_logarithm_firstorder_90Percentile+

-0.177*Skewness_logarithm_firstorder_Skewness+ -0.754*Skewness_squareroot_glcm_Imc2+

-0.575*Skewness_wavelet-HLH_gldm_SmallDependenceLowGrayLevelEmphasis+

0.410*Skewness_wavelet-LLL_glszm_LargeAreaHighGrayLevelEmphasis+ 0.551*Kurtosis_gradient_ngtdm_Coarseness+ 0.419*Kurtosis_Localbinarypattern2D_glcm_DifferenceEntropy+

-0.302*Kurtosis_Localbinarypattern2D_glrlm_RunEntropy+

0.579*Kurtosis_Localbinarypattern2D_glrlm_RunLengthNonUniformityNormalized+

-0.289*Kurtosis_wavelet-LLH_glcm_DifferenceEntropy+ -0.341*Kurtosis_wavelet-LHL_glcm_Imc1+

-0.983*Kurtosis_wavelet-LHL_gldm_DependenceEntropy+

-0.376*Kurtosis_wavelet-LHH_gldm_LargeDependenceEmphasis+

0.397*Kurtosis_wavelet-HLL_glrlm_ShortRunEmphasis+ 0.391*Kurtosis_wavelet-HLH_glcm_SumEntropy+

0.045*Mean_wavelet-LHL_ngtdm_Complexity+ -0.514

**PRM-Score**= 4.898*Radscore of Model 3+ 15.314*Ki-67+ -3.704*lymphovascular invasion+ -13.641

**GPRM-Score**= 8.309*Radscore of Model 3+ -0.686*Ki-67+ -21.46*lymphovascular invasion+

-676.6*MED23_VAF+ -84.12*REL_VAF+ 18.82


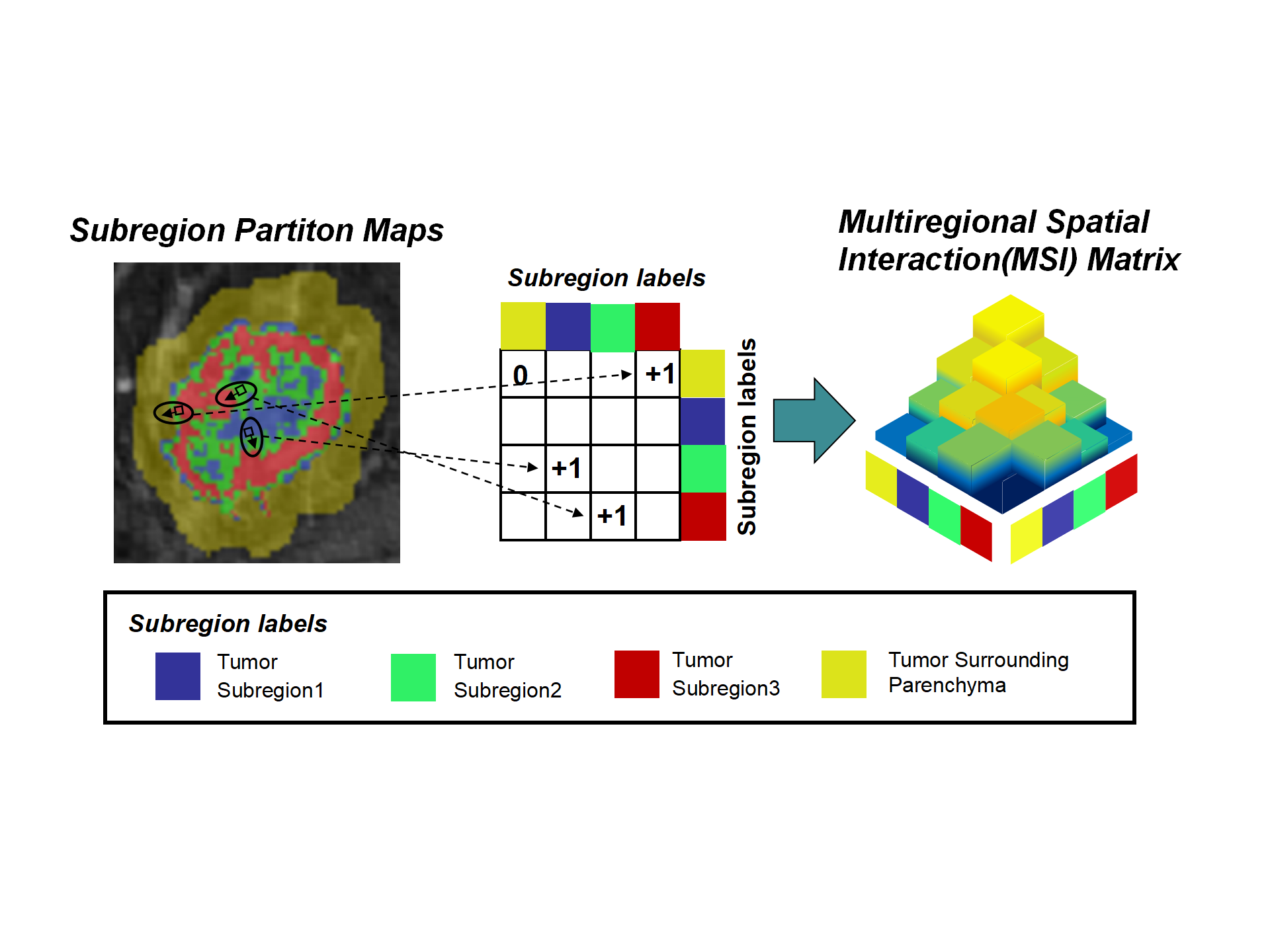


Figure S1. The illustration shows the use of a multiregional spatial interaction (MSI) matrix derived from tumor partition maps.

| Feature Type | Number | Meaning |
| --- | --- | --- |
| First order, absolute counts based on MSI matrix | 9 | Volume of each subregion (diagonal) and borders of two differing subregions (off-diagonal) |
| First order, relative counts based on normalized MSI matrix | 9 | Proportion of each subregion and borders of two differing subregions |
| Second order, contrast, homogeneity, correlation, and energy based on normalized MSI matrix | 4 | Summary statistics of spatial heterogeneity of intratumoral subregion maps |

Table S1. Twenty-two quantitative imaging features were extracted from the MSI matrix to measure spatial heterogeneity.

| **List of genes** **with mutations** | | | | | | | | | |
| --- | --- | --- | --- | --- | --- | --- | --- | --- | --- |
| AKT1 | BCL2A1 | CNR1 | FGFR2 | IGF1R | MAP3K1 | MMP9 | PGR | PTK6 | SRC |
| AKT3 | BCL2L2 | COL1A1 | FGFR3 | IKBKB | MAP3K13 | MSH2 | PHF6 | PTPN22 | SRMS |
| BRCA1 | BCOR | COL2A1 | FGFR4 | IL2 | MAP3K2 | MST1R | PHKA2 | PTPRD | STAG2 |
| BRCA2 | BCR | CREBBP | FGR | IL7R | MAP3K4 | MYB | PI4K2A | RAC1 | STAT1 |
| CDK4 | BIRC2 | CSF1R | FLT1 | INSR | MAP3K5 | NCOA1 | PI4KB | RAF1 | STAT5A |
| CDK6 | BIRC3 | CSF3R | FLT3 | INSRR | MAP3K6 | NCOR1 | PIK3C3 | RASAL1 | STAT5B |
| EGFR | BLK | CSNK1E | FLT4 | IRS1 | MAP3K7 | NEK2 | PIK3CB | RB1 | STK11 |
| ESR1 | BMX | CSNK2A1 | FOXA1 | ITCH | MAP4 | NF1 | PIK3CD | REL | SYK |
| mTOR | BRAF | CTCF | FOXO3 | ITGB3 | MAP4K3 | NF2 | PIK3CG | RET | TBL1XR1 |
| PIK3CA | BTK | CUX1 | FOXP1 | ITK | MAP4K4 | NFKB1 | PIK3R1 | RFC1 | TBX3 |
| PTEN | BUB1B | CYP19A1 | FRK | JAK1 | MAP4K5 | NFKB2 | PIK3R2 | RHOA | TEC |
| TP53 | CACNA1D | CYP2C8 | FYN | JAK2 | MAP7 | NFKBIA | PIKFYVE | RICTOR | TEK |
| ABCB1 | CAMK2G | CYP2C9 | GAB2 | JAK3 | MAPK1 | NOS1 | PIM1 | RIPK1 | TEP1 |
| ABL1 | CASP8 | CYP3A4 | GATA3 | JUN | MAPK10 | NOS2 | PIWIL1 | ROCK1 | TERT |
| ADA | CBFB | DCAF4L2 | GNAS | KAT8 | MAPK13 | NOS3 | PKD1 | ROCK2 | TET2 |
| AFF2 | CBL | DGKG | GPR32 | KCNB2 | MAPK14 | NOTCH1 | PLCG1 | RPGR | TGFBR1 |
| AKAP3 | CBLB | DNMT1 | GPS2 | KCNJ12 | MAPK3 | NOTCH2 | PLK2 | RPS6KA1 | TLR4 |
| AKT2 | CCND1 | DNMT3A | GRIN2A | KCNJ14 | MAPK4 | NR1H2 | PLK3 | RPS6KA3 | TNK2 |
| ALDH1A1 | CCND2 | DNMT3B | GSK3A | KCNJ2 | MAPK6 | NR1H4 | PMS2 | RPS6KA5 | TNKS2 |
| ALK | CCND3 | DYRK1A | GSK3B | KCNJ3 | MAPK7 | NR3C1 | PNPLA3 | RPS6KB1 | TOP1 |
| ALOX5 | CCNE1 | ECT2L | HDAC1 | KCNJ5 | MAPK8 | NRAS | PPARA | RPTOR | TOP2A |
| APC | CDC25A | EHMT2 | HDAC2 | KCNJ6 | MAPRE2 | NTRK1 | PPARG | RRM1 | TOP2B |
| APP | CDC25B | EPAS1 | HDAC4 | KCNJ9 | MAPRE3 | NTRK2 | PRDM1 | RRM2 | TOP3A |
| ARAF | CDC25C | EPHA1 | HDAC5 | KDM6A | MAPT | OR2G3 | PREX2 | HIST1H2BC | |
| ARID1A | CDH1 | EPHA2 | HDAC6 | KDR | MAST2 | OR2L2 | PRKAA1 | TPRX1 | TSC1 |
| ARID1B | CDH16 | EPHA3 | HDAC7 | KIF11 | MCL1 | PRKACA | RUNX1 | S1PR2 | TSC2 |
| ARID2 | CDK16 | EPHB1 | KIT | MDM2 | OR6A2 | PRKACB | RYR2 | SETD2 | TTK |
| ASXL1 | CDK2 | EPHB2 | KMT2B | MDM4 | PAK1 | PAK2 | PRKCA | SF3B1 | TYK2 |
| ATM | EPHB4 | HDAC9 | HIST1H1C | KMT2C | MECOM | PAK3 | PRKCB | SGK1 | TYRO3 |
| ATN1 | ERBB2 | HIF1A | TOP3B | KMT2D | MED23 | PALB2 | PRKCD | SGK2 | USH2A |
| ATP2B2 | CDKN1B | ERBB3 | HIST1H3B | KRAS | MEN1 | PARP1 | PRKCE | SHC1 | USP9X |
| ATR | CDKN2A | ERBB4 | HK2 | LCK | MERTK | PARP2 | PRKCG | SHH | WEE1 |
| ATRX | CETP | ERCC4 | HK3 | LPL | MET | PBRM1 | PRKCQ | SIK2 | WNK3 |
| AURKA | CHEK1 | FAAH | HMGCR | LYN | MICA | PDE4B | PRKCZ | SIRT1 | WNT7A |
| AURKB | CHEK2 | FAM47C | HRAS | MAP1A | MLH1 | PDE4C | PRKDC | SIRT7 | XBP1 |
| AURKC | CHRNA4 | FASN | HSP90AA1 | MAP1B | MLLT4 | PDE4D | PRKG1 | SLC5A1 | XDH |
| AXIN1 | CHUK | FBXW7 | HSP90AB1 | MAP2 | MMP1 | PDGFB | PRKX | SMAD4 | YES1 |
| AXL | CIC | FER | HSP90B1 | MAP2K1 | MMP14 | PDGFRA | PTGS2 | SMARCA4 | ZAP70 |
| BAK1 | CLEC19A | FES | HSPA4 | MAP2K2 | MMP2 | PDGFRB | PTK2 | SMO | ZFP36L1 |
| BAX | CNOT3 | FGFR1 | IGF1 | MAP2K4 | MMP3 | PDPK1 | PTK2B | SPEN | ZHX2 |

Table S2-1. List of genes with mutations detected by the 511-gene panel.

| **List of genes with copy number variations** | | | | | | | | | |
| --- | --- | --- | --- | --- | --- | --- | --- | --- | --- |
| ERBB2 | CAV3 | CHML | EXO1 | IRF4 | KLLN | NR2F2 | PRMT2 | RGMB | BCL2 |
| FGFR1 | CCL1 | CRIP1 | FANCD2 | IRS2 | MDM4 | OGG1 | PTPRN2 | S100B | TPMT |
| FGFR2 | CCL2 | CRK | FH | ITGB4 | MECP2 | OPCML | PVT1 | SLC19A3 | TRIM47 |
| PTEN | CCL20 | CTCFL | FOXQ1 | ATM | MIEN1 | PCID2 | RAB25 | SMYD3 | TRIM65 |
| MYC | CCL7 | CUL4A | FSIP1 | KAT6A | MTAP | PDGFRA | RAC2 | SOCS2 | TRPS1 |
| RB1 | CCL8 | DEK | GHRL | KDM1B | MTHFR | PEG10 | RAD18 | SOX9 | TUSC5 |
| AKT3 | ANKRD11 | DNMT1 | GLIS3 | KDM4A | MTOR | PFKFB3 | RAD50 | SSTR5 | UNC13D |
| CCND1 | CCNE1 | DUSP7 | GOLPH3L | KDM5A | NFIB | PIK3CA | RAD51 | ST8SIA4 | VHL |
| JAK2 | CD274 | EGFR | GRB7 | KDR | NOTCH1 | PIK3R1 | RBM17 | TKTL1 | VPS4B |
| TP53 | CDKN2A | EPSTI1 | IL15RA | KIF26B | NOTCH2 | PPAPDC1B | RECQL5 | TLR9 | WHSC1L1 |
| BMP7 | CDKN2B | ESR1 | IRAK1 | KIT | NOTCH3 | PRDM1 | RET | TNFRSF11A | ZBP1 |

Table S2-2. List of genes with copy number variations detected by the 511-gene panel.

| Characteristics | Training Cohort (n = 223) | | Validation Cohort (n = 92) | | P Value |
| --- | --- | --- | --- | --- | --- |
| Mean ± SD | 48.02±11.46 |  | 48.80±11.39 |  |  |
| Menopausal status |  |  |  |  | 0.761 |
| Menopausal | 106 | 47.5 | 50 | 54.3 |  |
| Premenopausal | 117 | 52.5 | 42 | 45.7 |  |
| Ki-67 status |  |  |  |  | 0.446 |
| <20% | 6 | 2.7 | 4 | 4.3 |  |
| ≥20% | 217 | 97.3 | 88 | 95.7 |  |
| Surgery type |  |  |  |  | 0.070 |
| Breast conservation | 43 | 19.3 | 10 | 10.9 |  |
| Mastectomy | 180 | 80.7 | 82 | 89.1 |  |
| T stage |  |  |  |  | 0.582 |
| 1 | 28 | 12.6 | 10 | 10.9 |  |
| 2 | 137 | 61.4 | 51 | 55.4 |  |
| 3 | 35 | 15.7 | 18 | 19.6 |  |
| 4 | 23 | 10.3 | 13 | 14.1 |  |
| N stage |  |  |  |  | 0.671 |
| 0 | 49 | 22.0 | 18 | 19.6 |  |
| 1 | 108 | 48.4 | 48 | 52.2 |  |
| 2 | 34 | 15.2 | 10 | 10.9 |  |
| 3 | 32 | 14.4 | 16 | 17.4 |  |
| Lymphovascular invasion |  |  |  |  | 0.782 |
| Present | 62 | 27.8 | 65 | 70.7 |  |
| Absent | 161 | 72.2 | 27 | 29.3 |  |
| Pathological type |  |  |  |  | 0.601 |
| IDC | 218 | 97.8 | 89 | 96.7 |  |
| ILC, IMPC | 5 | 2.2 | 3 | 3.3 |  |
| pCR |  |  |  |  | 0.703 |
| Yes | 90 | 40.4 | 35 | 38.0 |  |
| No | 133 | 59.6 | 57 | 62.0 |  |
| Recurrence or metastasis |  |  |  |  | 0.005 |
| Event | 36 | 16.1 | 28 | 30.4 |  |
| No event | 187 | 83.9 | 64 | 69.6 |  |

Table S3. The clinicopathological and prognostic characteristics of TNBC patients in the training set and validation set.

TNBC = triple-negative breast cancer; IDC = invasive ductal carcinoma; ILC = invasive lobular carcinoma; IMPC = invasive micropapillary carcinoma; pCR = pathological complete response.


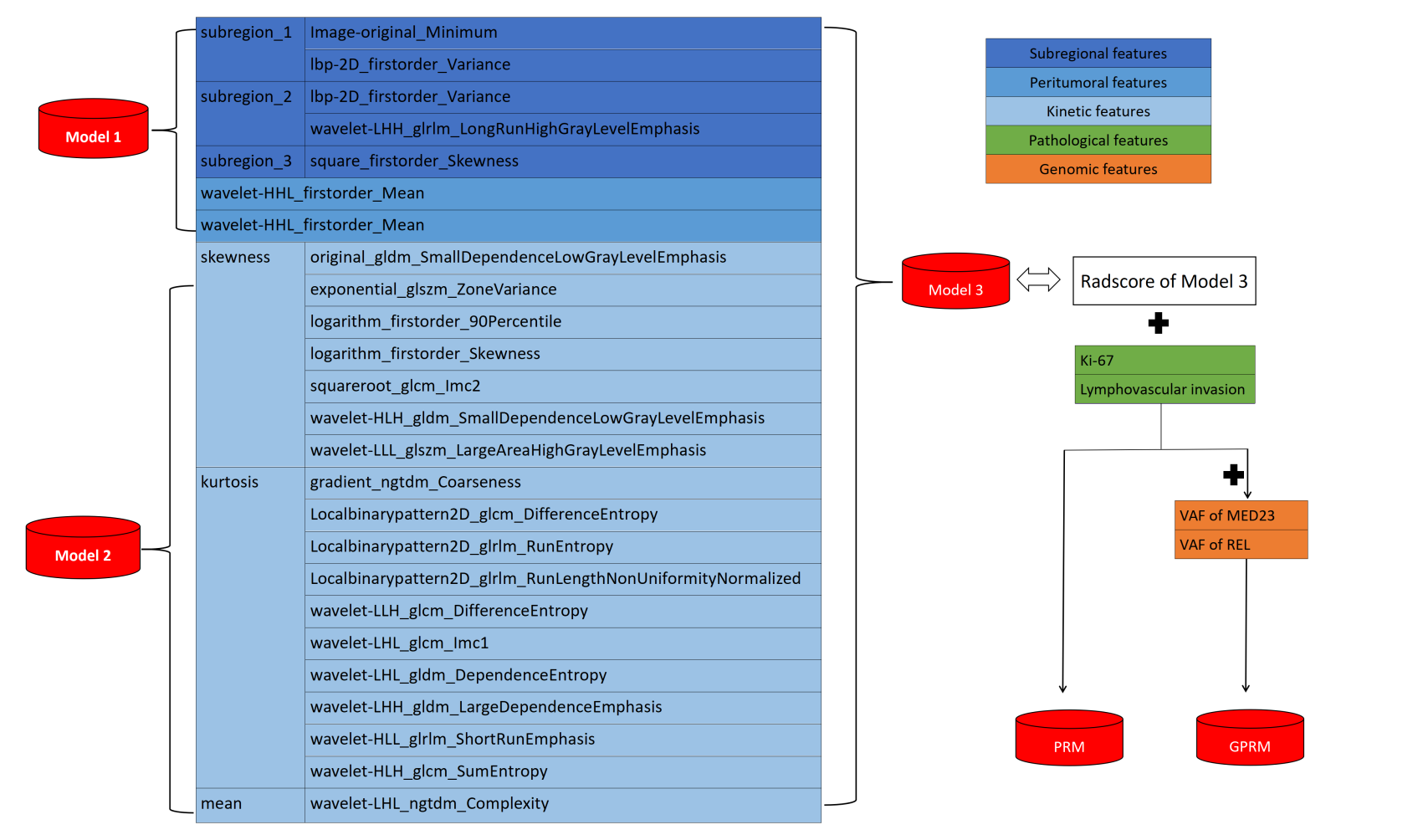


Figure S2. Specific features used to construct the models.

Model 1 was a radiomic model constructed by features from subregions and peritumoral regions; Model 2 was a radiomic model constructed by kinetic features from the tumor body; Model 3 was a radiomic model integrating features from Model 1 and Model 2; PRM = pathology-radiomics model; GPRM = genomics-pathology-radiomics model.

**References**

[1] Avants BB, Epstein CL, Grossman M, Gee JC. Symmetric diffeomorphic image registration with cross-correlation: evaluating automated labeling of elderly and neurodegenerative brain. Med Image Anal 2008; 12: 26-41.

[2] Sled JG, Zijdenbos AP, Evans AC. A nonparametric method for automatic correction of intensity nonuniformity in MRI data. IEEE Trans Med Imaging 1998; 17: 87-97.

[3] Wu S, Berg WA, Zuley ML et al. Breast MRI contrast enhancement kinetics of normal parenchyma correlate with presence of breast cancer. Breast Cancer Res 2016; 18: 76.

[4] Kim SY, Cho N, Shin SU et al. Contrast-enhanced MRI after neoadjuvant chemotherapy of breast cancer: lesion-to-background parenchymal signal enhancement ratio for discriminating pathological complete response from minimal residual tumour. Eur Radiol 2018; 28: 2986-2995.

[5] Thompson CM, Mallawaarachchi I, Dwivedi DK et al. The Association of Background Parenchymal Enhancement at Breast MRI with Breast Cancer: A Systematic Review and Meta-Analysis. Radiology 2019; 292: 552-561.

[6] Mercado CL. BI-RADS update. Radiol Clin North Am 2014; 52: 481-487.

[7] Wu J, Cao G, Sun X et al. Intratumoral Spatial Heterogeneity at Perfusion MR Imaging Predicts Recurrence-free Survival in Locally Advanced Breast Cancer Treated with Neoadjuvant Chemotherapy. Radiology 2018; 288: 26-35.
